# Successful 30-Day Nirmatrelvir/Ritonavir Treatment of a Patient Who Developed Multi-relapsed COVID-19 After Receiving R-CHOP Against Follicular Lymphoma

**DOI:** 10.7759/cureus.79019

**Published:** 2025-02-14

**Authors:** Kou Kimoto, Hitoshi Kawasuji, Yoshihiro Yoshida, Hiroshi Yamada, Shunsuke Yazawa, Hideki Tani, Yuki Koshiyama, Yoshimi Nabe, Shohei Kikuchi, Kentaro Nagaoka, Yoshitomo Morinaga, Yoshihiro Yamamoto

**Affiliations:** 1 Department of Clinical Infectious Diseases, Toyama University Graduate School of Medicine and Pharmaceutical Sciences, University of Toyama, Toyama, JPN; 2 Department of Microbiology, Toyama University Graduate School of Medicine and Pharmaceutical Sciences, University of Toyama, Toyama, JPN; 3 Department of Virology, Toyama Institute of Health, Imizu, JPN; 4 Department of Hematology, University of Toyama, Toyama, JPN

**Keywords:** bf.13, covid-19, multi-relapse, nirmatrelvir/ritonavir, r-chop

## Abstract

A 58-year-old male developed three coronavirus disease 2019 (COVID-19) relapses within three months after rituximab, cyclophosphamide, doxorubicin, vincristine, and prednisone (R-CHOP) chemoimmunotherapy against relapsed follicular lymphoma. Five- and 10-day remdesivir courses failed to achieve viral clearance. A 30-day nirmatrelvir/ritonavir course provided symptom resolution and sustained reverse transcription and quantitative polymerase chain reaction (RT-qPCR) negativity. Genome analyses identified cultured live virus (day 59) and nasopharyngeal-swab viral RNA (days 74, 82, 95) as Omicron BA.5 sublineage BF.13. Normal immunoglobulin (Ig)G levels and high neutralizing activities against BA.5 were maintained throughout the infection's course. Extended nirmatrelvir/ritonavir antiviral treatment may be effective for patients administered anti-CD20 therapy who develop prolonged/relapsed severe acute respiratory syndrome coronavirus 2 (SARS-CoV-2) infection despite possessing high neutralizing activities.

## Introduction

Exposure to severe acute respiratory syndrome coronavirus 2 (SARS-CoV-2) elicits a strong immunological innate and adaptive (both T and B cells) immune response [1]. Innate and adaptive immune populations work synergistically to prevent, limit, and clear SARS-CoV-2 infection, but immunocompromised patients, e.g., those with hematological malignancy, exhibit distinct alterations in SARS-CoV-2 susceptibility and infection and have shown poor coronavirus disease 2019 (COVID-19) outcomes, even in cases of the Omicron variant of SARS-CoV-2, due to their immunological deficiency and cancer-specific treatments [1-3].

Anti-CD20 antibodies (e.g., rituximab, obinutuzumab) are used to treat hematological malignancies to achieve B-cell depletion and impair humoral immune responses to SARS-CoV-2 [3]. The adaptive humoral immunity helps prevent SARS-CoV-2 infection and viral clearance [1], and thus patients who received anti-CD20 antibodies within the previous six months are at high risk for prolonged viral shedding, viral rebound, and chronic infection if they are infected with COVID-19 [4].

There are few reports of viral culture-confirmed prolonged and multi-relapsed COVID-19 in immunocompromised patients [5,6], and the utility of long-term antiviral therapy for persistent COVID-19 infection remains unclear [7]. We describe a case of multi-relapse COVID-19 after R-CHOP (rituximab, cyclophosphamide, doxorubicin, vincristine, and prednisone) chemoimmunotherapy following obinutuzumab maintenance therapy for follicular lymphoma. The patient required long-term antiviral therapy.

## Case presentation

A 58-year-old Japanese man with follicular lymphoma diagnosed in 2021 was treated with bendamustine plus obinutuzumab (six cycles, between June and November 2021) and then maintenance therapy with obinutuzumab (six cycles) until November 2022. He had not received SARS-CoV-2 vaccination. Follow-up CT revealed an enlarged abdominal periaortic lymph node, suggesting follicular-lymphoma relapse. R-CHOP chemotherapy was planned. However, he developed a fever and cough on November 26 (day 0) and tested positive at a follow-up examination (day 17) on quantitative reverse transcriptase-polymerase chain reaction (RT-qPCR) using nasopharyngeal swabs. Although he had no symptoms on day 17, he was treated with nirmatrelvir/ritonavir (five days) due to persistent nasopharyngeal viral RNA shedding (viral load 59 copies/μL).

**Figure 1:**
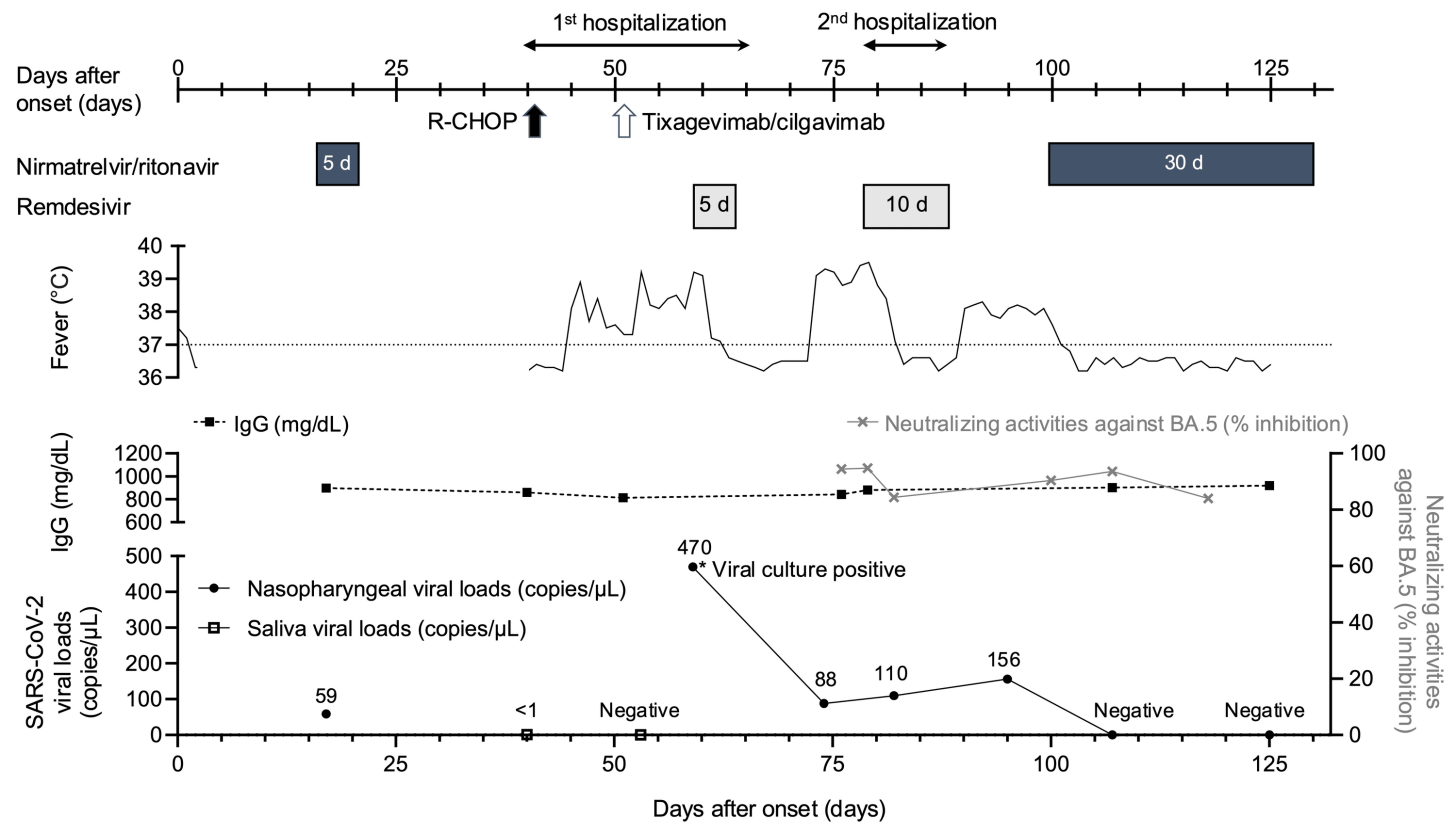
The clinical course Two courses of remdesivir (five and 10 days) failed to achieve viral clearance and cure, but the patient was successfully treated with an extended 30-day course of nirmatrelvir/ritonavir. The patient maintained normal IgG levels and high neutralizing activities against BA.5 throughout the course of the infection. R-CHOP: rituximab, cyclophosphamide, doxorubicin, vincristine, and prednisone.

Since the viral load in saliva screened at admission was <1 copies/μL (day 40), R-CHOP was started on day 41 (Figure 1). The patient developed high-grade fever and neutropenia on day 46 and was administered cefepime for febrile neutropenia. Considering the non-vaccinated status, post-exposure prophylaxis with tixagevimab/cilgavimab was administered by his attending physician (day 51).

**Figure 2:**
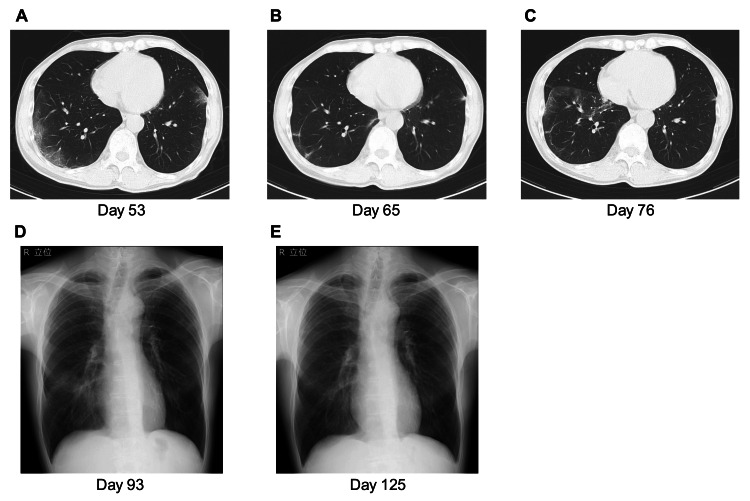
Chest computed tomography (CT) and X-ray images (A) Chest CT on day 53, in the first relapse, showing multiple bilateral ground-glass opacities (GGOs). (B) Chest CT on day 65, the first remission after five days of remdesivir, showing the resolution of most of the GGOs. (C) Chest CT on day 76, in the second relapse, showing GGO recurrence. (D) Chest X-ray on day 93, the third relapse, showing opacities in the right lower lung. (E) Chest X-ray on day 125, after 30 days of nirmatrelvir/ritonavir, showing resolution of the opacities.

CT scans (day 53) showed multiple bilateral ground-glass opacities (GGOs) (Figure 2A), but saliva SARS-CoV-2 RT-PCR testing was negative. Due to the persistent fever, the antimicrobial treatment was changed to meropenem, and micafungin was added. However, two sets of blood cultures taken before the change were negative, and the fever persisted even after neutrophil counts recovered (day 59).

Considering the possibility of lower sensitivity of RT-qPCR testing using saliva specimens, nasopharyngeal swabs were collected, and RT-qPCR and viral culture were performed (day 59). These revealed a nasopharyngeal viral load of 470 copies/μL and a positive viral culture, indicating the presence of culturable live virus. Based on the clinical course and GGOs similar to the typical CT findings of COVID-19 pneumonia (day 53) (Figure 2A), a first COVID-19 relapse was suspected. After the remdesivir initiation, the fever immediately resolved; remdesivir was discontinued at the standard five-day duration. Chest CT (day 65) showed improved GGOs (Figure 2B), and the patient was discharged. However, after discharge, he again developed a fever, and CT showed multiple new GGOs (day 76)(Figure 2C).

A second COVID-19 relapse was suspected. The patient was readmitted and remdesivir was re-administered for 10 days beginning on day 79. His fever disappeared immediately after the remdesivir's introduction, but three days after its completion, he developed a fever (third relapse). Chest X-rays revealed the reappearance of GGOs in the right lower lung (Figure 2D), and viral RNA was persistently detected by qRT-PCR using nasopharyngeal swabs (viral load: 156.2 copies/μL). Considering the need for longer-term viral suppression and the patient's refusal of prolonged hospitalization, nirmatrelvir/ritonavir was selected. An ethics review of off-label long-term nirmatrelvir/ritonavir use was sought; Toyama University Hospital's ethics committee approved it (approval no. MKTY2022034, March 10, 2023).

The fever immediately resolved after the nirmatrelvir/ritonavir initiation (day 100), and the GGOs on chest X-ray improved (Figure 2E). After two consecutive confirmations of negative nasopharyngeal qRT-PCR results, nirmatrelvir/ritonavir was discontinued after 30 days of treatment. No further COVID-19 relapses occurred.

The viral genome was analysed and neutralizing activities (NTs) were measured by the high-throughput chemiluminescence reduction neutralization test (htCRNT) during the clinical course. The live virus (day 59) and viral RNA extracted from nasopharyngeal swabs (days 74, 82, 95) were all identified as BF.13, an Omicron BA.5 sublineage. Because the pseudotype virus of BF.13 had not been generated, we measured the NTs against BA.5 as an alternative. The NTs against BA.5 using 100-fold diluted sera were high (>80% inhibition), and serum IgG was maintained at >800 mg/dL throughout the course (Figure 1).

RT-qPCR analysis

RT-qPCR using nasopharyngeal swabs or saliva was performed as described [8-11], or using the Ampdirect™ 2019-nCoV detection kit (Shimadzu, Kyoto, Japan) per the manufacturers' instructions. Quantification quality was controlled using AcroMetrix COVID-19 RNA Control (Thermo Fisher Scientific, Waltham, MA) or Exact Diagnostics SARS-CoV-2 standard (BioRad, Hercules, CA). The detection limit was ~0.4 copies/µL (two copies/5 µL).

Genome analysis

Whole-genome amplification of the viruses was performed using a modified version of the ARTIC Network protocol (Artic Network) for SARS-CoV-2 genome sequencing. A next-generation sequencing (NGS) library was constructed using the QIAseq FX DNA Library kit (Qiagen) and sequenced using the iSeq platform (Illumina, San Diego, CA). NGS reads were mapped to the SARS-CoV-2 Wuhan-Hu-1 reference genome sequence (GenBank accession no. MN908947.3) using the BWA-MEM algorithm (ver. 0.7.13-r1126). The variant-allele frequency analysis was conducted using VarScan ver. 2.4.3 [12].

Viral isolation from clinical specimens

VeroE6 cells overexpressing TMPRSS2 (VeroE6/TMPRSS2) (JCRB1819) obtained from the JCRB Cell Bank (Osaka, Japan) were grown in DMEM containing 10% heat-inactivated FBS, penicillin (50 units), and streptomycin/mL (50 µg). Nasopharyngeal swab specimens were suspended in PBS. Centrifugal supernatant of nasopharyngeal-swab suspension was added to VeroE6/TMPRSS2 cells seeded on a six-well plate and cultured (37°C, 5 days). The cytopathic effect was confirmed by visual observation/microscopy. All viral isolation procedures were performed in a University of Toyama biosafety level 3 laboratory.

Measurement of neutralizing activities using htCRNT assay

The measurement of NTs using sera was done as described [13,14]. Vesicular stomatitis virus (VSV) pseudotype bearing SARS-CoV-2 S protein was generated as described [15]. The pseudotyped VSVs were stored at −80°C until use. Each sample's neutralizing effects against pseudotyped VSVs were examined by a htCRNT as described [14,16]. Briefly, 100-fold diluted serum samples were co-incubated with pseudotyped SARS-CoV-2 for 1 hr. The pseudotyped viruses' infectivity to VeroE6/TMPRSS2 cells (JCRB1819) was determined by measuring the luciferase activity 24 hr later. Samples without pseudotyped virus and those with pseudotyped virus but not serum were defined as positive (0%) and negative (100%) infection-neutralizing controls, respectively.

## Discussion

We described a patient who developed prolonged infective SARS-CoV-2 Omicron-variant BF.13 COVID-19 relapses after R-CHOP chemotherapy for a follicular lymphoma relapse, following prior maintenance therapy with obinutuzumab. Whole-genome sequencing revealed the persistence of the infecting lineage BF.13. Viral shedding continued for more than three months, with confirmed viral infectivity on day 59.

The COVID-19 recurrence may have been triggered by the R-CHOP therapy on day 41 after the initial COVID-19 episode, and a total of three relapses occurred despite the preservation of IgG level and NTs. Nakamura et al. described a similar case of recurrent COVID-19 caused by SARS-CoV-2 Omicron variant BA.1.1.2 in a 79-year-old male with diffuse large B-cell lymphoma who received R-CHOP 11 days after a first COVID-19 episode [6]; however, this case was seronegative. The prolonged and relapsing course of COVID-19 during B-cell depletion therapy is largely attributed to impaired viral clearance due to diminished humoral immunity and is generally considered to occur in a seronegative state. However, the IgG level in the present case was not low (>800 mg/dL), and the NTs against BA.5 on days 76, 79, 82, 100, 107, and 118 were all as high as those in convalescent patients (>80% inhibition). These results indicated that sero-negativity was not the main cause of persistent infection. Persistent viral shedding and sero-negativity have been described as insufficient to explain the reasons for persistent SARS-CoV-2 infection because all SARS-CoV-2 infections in patients with B-cell depletion have led to a protracted clinical course [17]; in that report, the patients with persistent SARS-CoV-2 infection had abnormal or dysregulated T- cell responses, which does not lead to the virus' eradication [17]. Cellular immunosuppression induced by CHOP chemotherapy may have additionally contributed to the impaired immune clearance mechanism. 

In addition to R-CHOP potentially triggering the relapse, prior anti-CD20 antibody therapy before COVID-19 infection also poses a risk. In this case, obinutuzumab was administered 22 days prior to infection. Parra et al. described nine patients of 52 (17%) who experienced recurrence or relapse following anti-CD20 therapy [4]; all relapsed patients received anti-CD20 therapy during the 6 months before COVID-19. Similar to the finding that peripheral B-cell depletion induced by rituximab lasts ≥6 months [18], patients who received anti-CD20 therapy during the six months before COVID-19 infection require close attention to COVID-19 recurrence.

We selected an extended nirmatrelvir/ritonavir regimen, and the 30 days of nirmatrelvir/ritonavir finally cleared the viral secretion and produced symptom resolution. Our patient also tolerated the long-term nirmatrelvir/ritonavir with no significant adverse events or drug-drug interactions, as in another study [19]. An extended nirmatrelvir/ritonavir course may be an option for patients who received anti-CD20 therapy and then developed prolonged and relapsed SARS-CoV-2 infections despite possessing high NTs. Further studies must investigate the best therapeutic strategies for persistent COVID-19 infection.

## Conclusions

We describe a case of multi-relapse COVID-19 after R-CHOP chemoimmunotherapy following obinutuzumab maintenance therapy for follicular lymphoma. Five- and 10-day remdesivir courses failed to achieve viral clearance, although normal IgG levels and high NTs against BA.5 were maintained throughout the infection's course. A 30-day nirmatrelvir/ritonavir course finally provided symptom resolution and sustained RT-qPCR negativity. Extended nirmatrelvir/ritonavir antiviral treatment may be an option for patients who received anti-CD20 therapy and then developed prolonged and relapsed SARS-CoV-2 infections despite possessing high NTs.
